# P30 A case of recurrent orbital myositis

**DOI:** 10.1093/rap/rkab068.029

**Published:** 2021-10-19

**Authors:** Annie Dixey, Daniele Lorenzano, Alice Mason

**Affiliations:** UHS, Southampton, United Kingdom

## Abstract

**Case report - Introduction:**

An interesting case of an 11-year recurrent isolated lateral rectus myositis. Initially responsive to short courses of oral steroids; however, this has more recently become refractory and now the patient is unable to wean off steroids. The use of biologics in these patients is uncommon and there are very few case reports of this and the outcomes. We present a case of refractory orbital myositis treated with rituximab.

**Case report - Case description:**

A 38-year-old female presented in 2009 with ocular pain, swelling and diplopia. After review by the ophthalmology team an MRI orbits was requested which showed swelling and enhancement of the right lateral rectus muscle, consistent with orbital myositis. She had no other symptoms of myositis elsewhere, and no other symptoms suggestive of a connective tissue disease. Her ANA, ENA and extended myositis panel were negative and therefore aetiology was uncertain. She had no past medical history, but was later diagnosed with hypothyroidism with positive TPO antibodies. Throughout the 11 years since diagnosis her thyroid disease has been well controlled, and she has had normal free T3/T4 and TSH. It was felt that thyroid eye disease was unlikely to present with a single muscle.

She was initially treated with a course of 40mg oral prednisolone reducing over 6 weeks and symptoms resolved. In 2013 she had her first relapse, which was treated with prednisolone reducing over the course of 12months, and again symptoms resolved. Her third relapse in 2019 was treated with steroids, but patient was unable to wean off this, requiring 60mg of prednisolone, and at this point was referred to rheumatology for steroid sparing agents. Between 2013 and 2019 there was MRI progression with volumetric enlargement of the right lateral rectus with intra-orbital space reduction.

Under the care of rheumatology, she had repeat connective tissue disorders and myositis screen. She was started on azathioprine as a steroid sparing agent. This had little effect and she was still debilitated by orbital pain and diplopia, and unable to reduce her prednisolone below 30mg daily. She trialled intra-orbital steroid injections with little benefit. She has now been started on rituximab infusions to allow us to wean steroids. Thus far she has had two doses and we await the outcome from this treatment.

**Case report - Discussion:**

Orbital myositis can be a debilitating condition causing diplopia and pain, as in the case of this 38-year-old female who has been off work for the past year due to the condition. It can affect single muscles or multiple muscles and may be unilateral or bilateral. The major differential diagnosis is thyroid eye disease which would not usually cause an isolated myopathy, is usually painless and slowly progressive and as such was felt unlikely to be the underlying pathology in this case.

From a literature review into idiopathic orbital inflammation, including myositis, 75% of patients are found to have a good response to corticosteroids. Second-line treatments include radiotherapy, methotrexate/azathioprine and other biologic agents.

A case report in 2014 of 10 patients with orbital myositis refractory to steroids and at least one other immunosuppressant, demonstrated that rituximab was safe and effective, with 7/10 patients noting improvement of their symptoms. Out of those seven patients, four had been on steroids at induction of rituximab and all of the patients were able to reduce their steroid dose. The patients in this trial received two initial doses of rituximab and were permitted to have a further dose at 24 weeks if there were recurrence of symptoms. Four out of the seven patients required a further infusion after 24 weeks.

In a review of the literature, we have noted a further two case reports of the use of rituximab in orbital myositis from 2008 and 2012 with good response. Both patients were unable to wean from corticosteroids and had tried other DMARDs such as methotrexate. Other case reports of biologics therapy include a case report of two patients in which adalimumab was used and allowed steroid reduction with good results for at least 9 months.

**Case report - Key learning points:**

- Orbital myositis should be assessed for other features of polymyositis, as well as investigated for thyroid eye disease
- Most patients will have a good response to oral corticosteroids
- Around a quarter of cases can have refractory disease. In these patients there remains a management dilemma. Options include radiotherapy, disease modifying agents such as methotrexate and azathioprine, and biologic therapy such as adalimumab or rituximab
- There is limited evidence in the literature as to the best treatment option and ideal length of treatment for refractory orbital myositis cases

